# Supplementary material for: Intensive vital signs monitoring reduces 30-day mortality among stroke patients: A cohort study from Tanzania
Source: PLoS One. 2025 Jul 21;20(7):e0328710. doi: 10.1371/journal.pone.0328710 (PMC12279090; doi:10.1371/journal.pone.0328710)
Supplement: S1 Appendix — (PDF) [file pone.0328710.s001.pdf]

S1_Appendix: Case Report Form (English Version)

Vital signs monitoring and stroke outcomes_Tumaini et al (2023/24)

Questionnaire No: ___________________

PART 1: PATIENT'S IDENTIFICATION

Hospital No: ___________________
Age (years): ___________________
Sex: Male / Female (Circle applicable)
Address: ___________________
Phone Number (Patient): ___________________
Phone Number (Close Relative): ___________________

PART 2: CLINICAL HISTORY

Date and time of Stroke Onset: ________________ ________________

Type of Stroke (from medical records):

☐ Ischemic

☐ Hemorrhagic

☐ Other (specify) ___________________

Do you have any of the following medical conditions?

☐ Hypertension

☐ Diabetes Mellitus

☐ Chronic Kidney Disease

☐ Heart Disease

☐ HIV Infection

Are you currently on any of the following medications?

☐ Antihypertensives

☐ Antidiabetics

☐ Lipid-lowering drugs

☐ Antiplatelets/Anticoagulants

☐ Others (specify) ___________________

PART 3: BEHAVIORAL AND LIFESTYLE HISTORY

Do you currently smoke?

☐ Yes

☐ No

If Yes, for how long? _______ years
How many cigarettes per day? _______

Did you smoke in the past?

☐ Yes

☐ No

If Yes, when did you stop? _______

Do you drink alcohol?

☐ Yes

☐ No
f Yes, what type? ___________________
How much per week? ___________________ (calculate units of alcohol per week)

Level of Physical Activity (Assessed via International Physical Activity Questionnaire - IPAQ Short Form):
☐ Low
☐ Moderate
☐ Vigorous

PART 4: EXAMINATION FINDINGS

Vital Signs Monitoring (Every 6 or 12 Hours from Admission to 72 Hours)

Time (Hours)	Systolic BP (mmHg)	Diastolic BP (mmHg)	Pulse Rate (bpm)	Respiratory Rate (cycles/min)	Oxygen Saturation (%)	Temperature (°C)	Blood Glucose (mmol/L)
Admission	_______	_______	_______	_______	_______	_______	_______
6	_______	_______	_______	_______	_______	_______	_______
12	_______	_______	_______	_______	_______	_______	_______
18	_______	_______	_______	_______	_______	_______	_______
24	_______	_______	_______	_______	_______	_______	_______
30	_______	_______	_______	_______	_______	_______	_______
36	_______	_______	_______	_______	_______	_______	_______
42	_______	_______	_______	_______	_______	_______	_______
48	_______	_______	_______	_______	_______	_______	_______
54	_______	_______	_______	_______	_______	_______	_______
60	_______	_______	_______	_______	_______	_______	_______
66	_______	_______	_______	_______	_______	_______	_______
72	_______	_______	_______	_______	_______	_______	_______

PART 5: STROKE SEVERITY AND FUNCTIONAL OUTCOMES

National Institutes of Health Stroke Scale (NIHSS) Score:
At Admission: _______
At 72 Hours: _______
At 14 Days: _______
At 30 Days: _______

Modified Rankin Scale (mRS) Score:
At Admission: _______
At 72 Hours: _______
At 14 Days: _______
At 30 Days: _______

PART 6: LABORATORY FINDINGS (AT ADMISSION)

Laboratory Test	Result	Reference Range

Hemoglobin (g/dL) _______ M: 13.2-16.5, F: 11.5-16.0

WBC Count ($\times 10^9$/L) _______ 4.0-11.0

Platelet Count ($\times 10^9$/L) _______ 150-450

Serum Sodium (mmol/L) _______ 135-145

Serum Potassium (mmol/L) _______ 3.5-5.0

Serum Creatinine (mmol/L) _______ 50-110

Total Cholesterol (mmol/L) _______ <5.2

LDL Cholesterol (mmol/L) _______ <3.0

HDL Cholesterol (mmol/L) _______ >1.0

Triglycerides (mmol/L) _______ <1.7

PART 7: IMAGING FINDINGS

Brain Imaging Performed?
☐ Yes
☐ No

If Yes, Type of Imaging:
☐ CT Scan
☐ MRI
☐ Other (specify) ____________________
Findings:
____

Stroke Subtype:
☐ Ischemic Stroke
☐ Hemorrhagic Stroke
☐ Other (specify) ____________________

CONCLUSION

This structured interview tool is designed to collect comprehensive patient information regarding vital signs monitoring and outcomes among stroke patients. Data will be used to assess trends in vital sign variations, functional outcomes, and mortality rates in both the intensive (6-hourly) and standard (12-hourly) monitoring groups.

Thank you for your participation.
