# Supplementary material for: Intensive vital signs monitoring reduces 30-day mortality among stroke patients: A cohort study from Tanzania
Source: PLoS One. 2025 Jul 21;20(7):e0328710. doi: 10.1371/journal.pone.0328710 (PMC12279090; doi:10.1371/journal.pone.0328710)
Supplement: S1 Table — (DOCX) [file pone.0328710.s002.docx]

**S1 Table. Functional disability and mortality at 14 days among study participants by vital signs monitoring strategy**.

| Characteristic | Total  N=306 | Vital signs monitoring strategy | | p value |
| --- | --- | --- | --- | --- |
|  |  | 6-hourly  n=153 (50.0%) | 12-hourly  n=153 (50.0%) |  |
| **mRS at 14 days** |  |  |  |  |
| Mild disability | 21 (6.9%) | 9 (5.9%) | 12 (7.8%) |  |
| Moderate disability | 32 (10.5) | 14 (9.2) | 18 (11.8) |  |
| Moderate to severe disability | 83 (27.1) | 41 (26.8) | 42 (27.5) | 0.848 |
| Severe disability | 94 (30.7) | 50 (32.7) | 44 (28.8) |  |
| Death | 76 (24.8%) | 39 (25.5%) | 37 (24.2%) |  |

mRS: the modified Rankin Scale
