# Supplementary material for: Intensive vital signs monitoring reduces 30-day mortality among stroke patients: A cohort study from Tanzania
Source: PLoS One. 2025 Jul 21;20(7):e0328710. doi: 10.1371/journal.pone.0328710 (PMC12279090; doi:10.1371/journal.pone.0328710)
Supplement: S2 Table — (DOCX) [file pone.0328710.s003.docx]

**S2 Table. Independent predictors of 30-day mortality among study participants.**

| Variable | Relative risk (95% CI) | P value* |
| --- | --- | --- |
| Age ≥ 60 years | 1.25 (1.00–1.57) | **0.049** |
| Female sex | 0.96 (0.77–1.20) | 0.742 |
| Smoking history | 0.88 (0.65–1.18) | 0.383 |
| Current alcohol use | 1.40 (1.12–1.74) | **0.003** |
| Hypertension | 1.20 (0.89–1.60) | 0.231 |
| Diabetes mellitus | 0.85 (0.64–1.14) | 0.276 |
| Chronic kidney disease | 1.34 (0.90–2.00) | 0.151 |
| Heart disease | 1.60 (1.07–2.37) | **0.020** |
| HIV infection | 1.30 (0.77–2.18) | 0.321 |
| Intensive vital signs monitoring | 0.65 (0.53–0.79) | **<0.001** |
| Hemorrhagic stroke | 1.18 (0.92–1.50) | 0.186 |
| Baseline stroke severity: NIHSS ≥16 | 5.14 (3.71–7.11) | **<0.001** |

* P values in bold indicate statistical significance (P < 0.05).

CI, confidence interval; NIHSS, National Institutes of Health Stroke Scale.
