# Supplementary material for: Intensive vital signs monitoring reduces 30-day mortality among stroke patients: A cohort study from Tanzania
Source: PLoS One. 2025 Jul 21;20(7):e0328710. doi: 10.1371/journal.pone.0328710 (PMC12279090; doi:10.1371/journal.pone.0328710)
Supplement: S1 Fig — (PDF) [file pone.0328710.s004.pdf]

S1 Figure. Blood pressure variability during the first 72 hours post-admission by vital signs monitoring strategy.

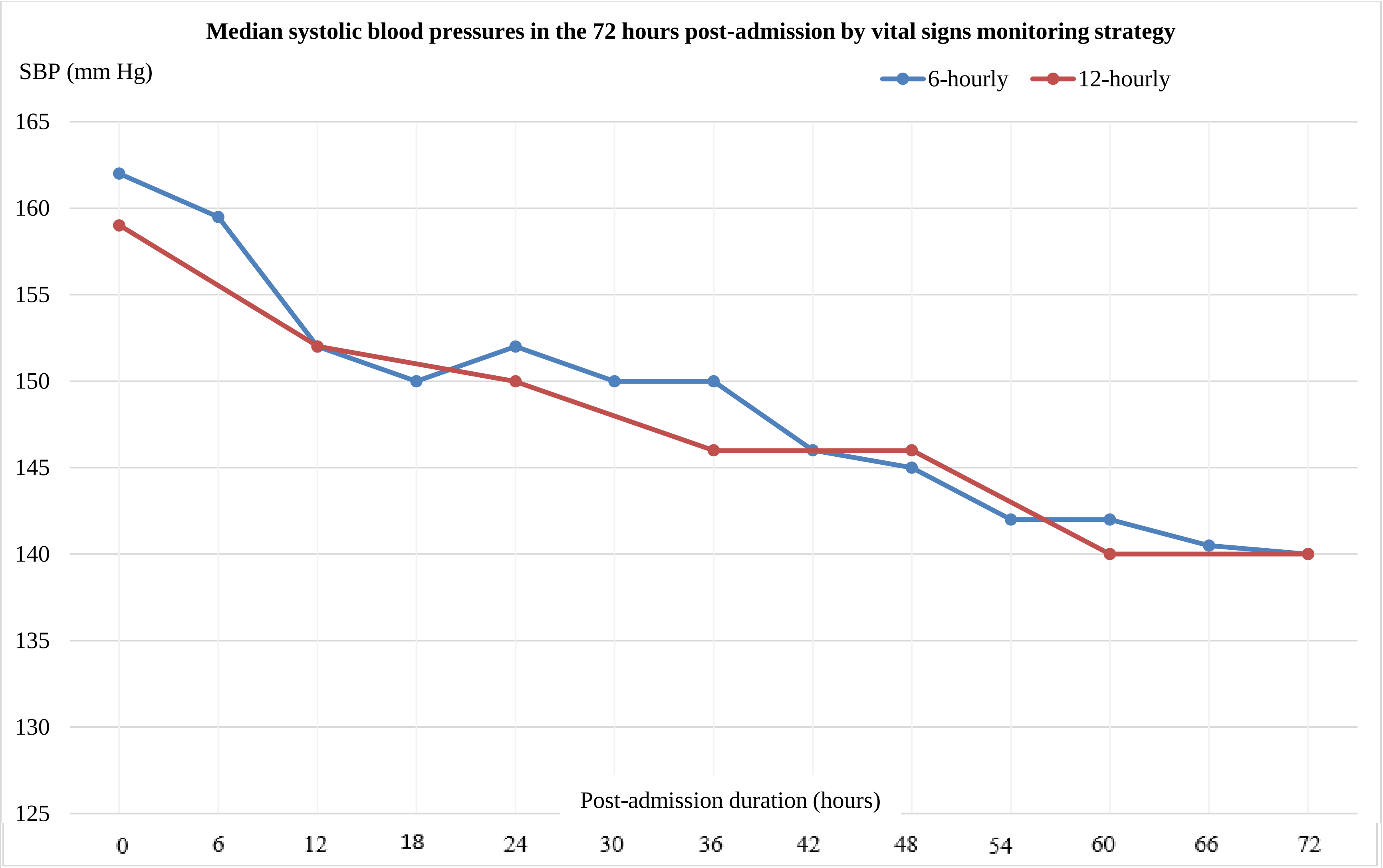

Systolic blood pressure variability revealed no clear differential trend between the vital signs monitoring strategies.

S1 Figure. Blood pressure variability during the first 72 hours post-admission by vital signs monitoring strategy.

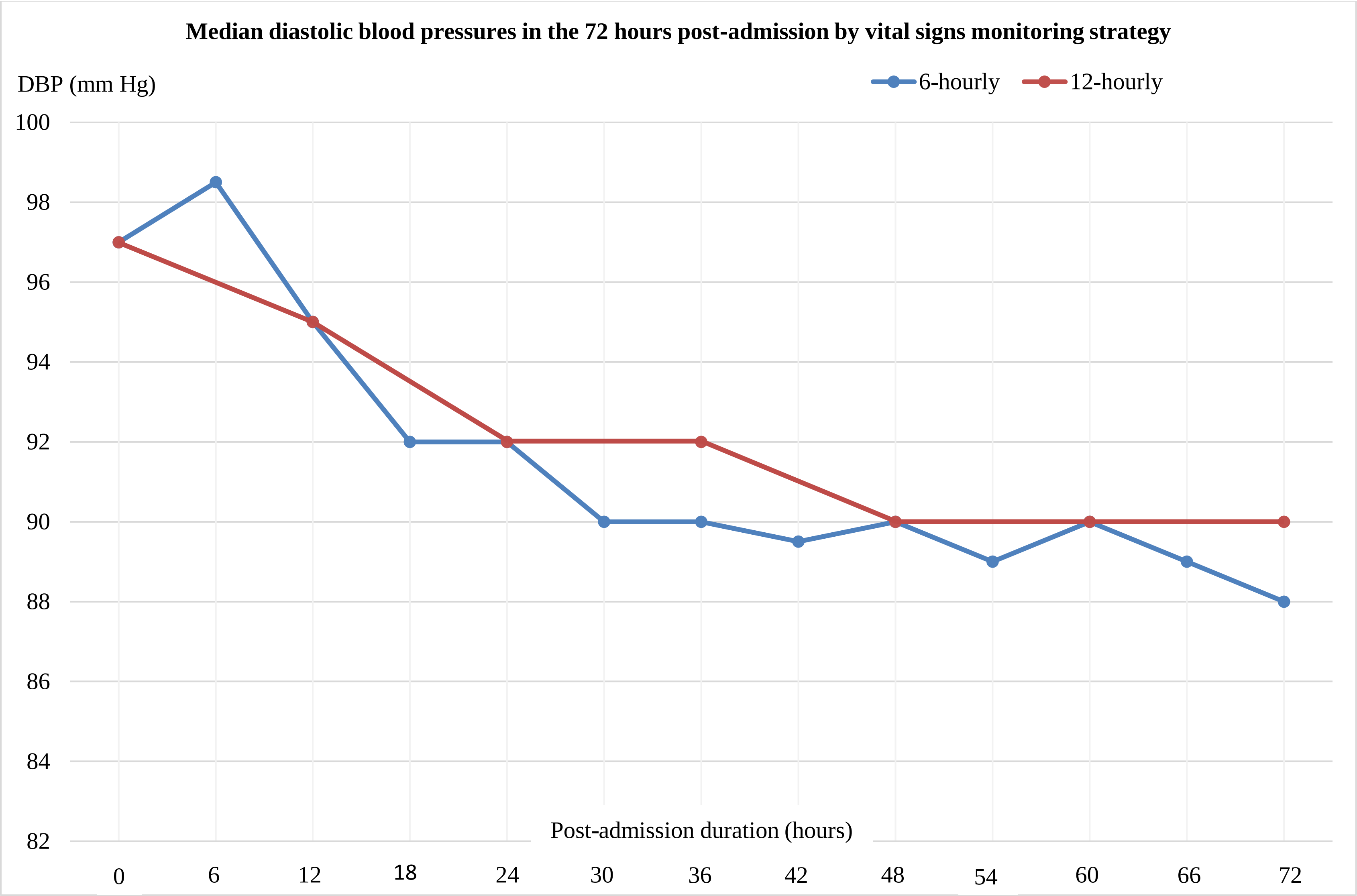

In the acute post-stroke period, patients monitored every 6 hours exhibited a more pronounced and sustained reduction in diastolic blood pressure compared to those monitored every 12 hours.
