# Supplementary material for: Intensive vital signs monitoring reduces 30-day mortality among stroke patients: A cohort study from Tanzania
Source: PLoS One. 2025 Jul 21;20(7):e0328710. doi: 10.1371/journal.pone.0328710 (PMC12279090; doi:10.1371/journal.pone.0328710)
Supplement: Attachment — Submitted filename: PLOS One Review_Mar 2025.docx [file pone.0328710.s005.docx]

PLOS One Review 3.19.2025

This is a very compelling study that supports the establishment of stroke units with advanced monitoring capabilities in resource-limited settings.

Abstract:

- In the background, there are a lot of parenthesis which is distracting to the reader. Consider removing the parentheses with outcomes as you can list them below in the methods.
- No need to say 306 patients twice, put in results only.
- In the results you mention diastolic blood pressure variability at 72 hours as a main finding but above say you are reporting on outcomes at 30 days. Also, you go beyond simply listing the result and interpret it when you say “underscoring its role in clinical stabilization”; consider removing the interpretation and putting this in the Conclusion instead.
- First sentence of the Conclusion restates the results almost verbatim.
- Excellent final sentence that makes a compelling argument and summarizes the study findings.

Introduction:

- This is extremely well written, concise, and beautifully organized. Well done! Just a few comments to improve it:
- One addition that could be helpful to this section would be listing the vital signs you are referring to. Is it heart rate, blood pressure, oxygen saturation? More?
- Lines 66-68 are basically restating what is above and I don’t think they are needed at all. The introduction ended beautifully at line 65 on the previous page. If you really wanted a final sentence remove the repeated info and leave only “…Hospital. The results of this study will contribute evidence-based recommendations for optimizing stroke care in LMICs.”

Methods:

- Was ethical approval obtained from NIMR, the National IRB of Tanzania, in addition to the Research and Publication Committee of Muhimbili? I believe studies in Tanzania need NIMR approval, too.
- Were the abnormal vitals reported to clinicians in real time? (line 77-78) Consider adding this detail.
- Lines 84-85. You do not need to re-state that studies indicated improved stroke outcomes as you already built the case well in the Introduction. Please remove.
- Line 85. Do not say “we wanted to evaluate” but rather say “we evaluated” as it is more direct, crisp writing.
- Line 91-92. Perhaps define stroke mimics and also reliable informant when describing exclusion criteria.
- In Sample Size, why was 35% relative reduction chosen?
- For the randomization section, why was it opened by the participant? In order to take the vital signs, I’m assuming a healthcare provider had to know if they were randomized to one group vs the other so they could take the vital signs on time, so how was it blinded?
- Line 161. How was follow-up performed? By calling participants or home visits or clinic follow-up visits?

Results:

- Overall excellent job summarizing the pertinent findings from the tables.
- Vitals signs at 72 hours by monitoring strategy – Was the intensive monitoring group just sicker, and that is why they had increased mortality? This should be discussed in Limitations or Discussion.
- There are so many tables and figures. Consider removing Table 5 that talks about outcomes at 14 days, or including it as a supplemental table, since there was nothing significant anyway. The more important results are the 30 day outcomes tables.
- Another way to cut down on the number of tables would be to combine tables 6 and 7 into an outcomes table to include both disability and mortality.

Discussion:

- I haven’t seen title headings for sections of this Discussion before, and don’t really like them. I’ll leave it up to the editor to decide if they will allow the authors to do this or not, but would advise against it.
- Lines 271-272. I think the interpretation here as the vital signs monitoring reduced diastolic blood pressure is incorrect. The monitoring didn’t reduce the blood pressure, but rather it just was that patients that had more intensive monitoring had lower BP. Please reword this inaccurate sentence. This is also where you can discuss if perhaps the intensive monitoring group were just sicker since they had lower diastolic BP. Also consider adding this to the Limitations.
- The whole vital signs monitoring paragraph needs to be re-thought as per above.
- The discussion of functional disability at 72 hours is good, but it doesn’t seem to be highlighted in the Intro/outcomes as a main outcome. Perhaps add it above. Also, consider discussing functional disability at 30 days.
- In the Conclusion paragraph, consider a sentence like that in the abstract that calls for the establishment of stroke units – this was very strong.
