# Supplementary material for: Intensive vital signs monitoring reduces 30-day mortality among stroke patients: A cohort study from Tanzania
Source: PLoS One. 2025 Jul 21;20(7):e0328710. doi: 10.1371/journal.pone.0328710 (PMC12279090; doi:10.1371/journal.pone.0328710)
Supplement: Attachment — Submitted filename: Response to Reviewers.docx [file pone.0328710.s007.docx]

**Response to Reviewers’ Comments**

**Journal Requirements**

Please ensure that your manuscript meets PLOS ONE's style requirements, including those for file naming.

**Response**: Thank you. We have reviewed the PLOS ONE formatting guidelines and revised the manuscript to meet the Journal's style requirements.

At this time, please upload the minimal data set necessary to replicate your study's findings to a stable, public repository (such as figshare or Dryad) and provide us with the relevant URLs, DOIs, or accession numbers that may be used to access these data.

*Response*: We appreciate the journal’s commitment to open science and transparency. In response to your request to upload the minimal dataset to a public repository, we respectfully provide the following revised Data Availability Statement, considering institutional policy and ethical constraints:

**Data Availability**

The dataset underlying this study contains sensitive personal and health-related information from a small number of participants at a single center. Although data were de-identified, there remains a risk of re-identification, particularly in a resource-limited setting. Public sharing of the dataset was not approved by the Muhimbili University of Health and Allied Sciences (MUHAS) Institutional Review Board (IRB) at the time of ethics approval.

However, the authors support data transparency and reproducibility. Qualified researchers may request access to the dataset through either of the following contacts:

1. **Corresponding Author**

Dr. Basil Tumaini

Email: [tumainibasil@gmail.com](mailto:tumainibasil@gmail.com)

Tel: +255 767 847 258

2. **Director of Research and Publications, MUHAS (IRB Contact)**

P.O. Box 65001, Dar es Salaam, Tanzania

Tel: +255-22-2152489

Email: drp@muhas.ac.tz

All requests will be reviewed in accordance with MUHAS policies and ethical guidelines.

**Reviewer #1**:

We thank Reviewer #1 for the constructive and insightful feedback. Below is our detailed point-by-point response:

Title: Ok

*Response*: Thank you.

Abstract: OK, provided the concerns in the methodology section below are addressed and reflected in the abstract in the corrected version

*Response:* We agree with the Reviewer. The Abstract has been revised to reflect clarifications in the Methods section, including the justification of the study design.

Introduction: OK

*Response*: Thank you.

**Methodology:**

Ethics: OK

*Response*: Thank you.

Study design: A randomized clinical trial would have been a better study design in this case. In fact, according to the descriptions given by the authors of the randomization and allocation of various study arms, it seems that a trial has indeed been conducted. Why the authors are calling it a cohort study is still unclear. The authors need to justify why this is a cohort study, and if that is the case, then why a randomized trial was not conducted.

*Response*: We sincerely thank the reviewer for this important observation. We have clarified in the revised manuscript that this was a prospective cohort study, not a randomized clinical trial (RCT). While random allocation was used to strengthen internal validity, the study does not meet key criteria for an RCT for the following reasons:

a. **Nature of the exposure:** The monitoring schedules (6-hourly vs. 12-hourly vital signs checks) were derived from routine hospital practice and reflected existing standards of care. No investigational treatments, medications, or procedures were introduced by the research team.

b. **Lack of blinding and protocol-driven interventions:** All healthcare providers and assessors were aware of group allocation, and treatment decisions—including medication, fluids, and investigations—were made independently by clinical teams, without influence from the study protocol.

c. **Study intent:** The study aimed to evaluate real-world clinical outcomes associated with existing monitoring practices, not to test the efficacy of a novel intervention under controlled conditions. This aligns with the principles of observational research.

d. **Ethical and operational considerations:** As both monitoring strategies represented acceptable care standards, the study received ethical approval as an observational cohort study embedded within hospital workflows.

We have revised the *Study design* section of the manuscript accordingly to reflect this clarification and have ensured consistency throughout the text.

Study population: ok

*Response*: Thank you.

Sample size: please elaborate how the sample size was calculated and what formula was used. It seems the formula used is also that of an RCT

*Response:* We thank the Reviewer for this important point. The sample size was calculated using a formula for comparing two proportions, a method appropriate for both randomized clinical trials and prospective cohort studies. The aim was to detect a relative reduction in 30-day mortality from 61% in the 12-hourly monitoring group to 37% in the 6-hourly monitoring group These estimates were based on mortality data from two published prospective cohort studies in sub-Saharan Africa: one conducted in Tanzania [1] reporting a 61% mortality rate, and another in Nigeria [2] reporting a 37% mortality rate among stroke patients.

The formula used was: *n = [(Zα/2 + Zβ)² × (p₁(1−p₁) + p₂(1−p₂))] / (p₁ − p₂)²*,

where: p₁ = 0.61 (expected mortality in the 12-hourly group), p₂ = 0.37 (expected mortality in the 6-hourly group), Zα/2 = 2.576 (for two-sided α = 0.01), Zβ = 1.28 (for 90% power).

We have revised the *Sample Size* section to include the formula and parameters.

References

1.	Matuja SS, Munseri P, Khanbhai K. The burden and outcomes of stroke in young adults at a tertiary hospital in Tanzania: a comparison with older adults. BMC Neurology. 2020;20:1-10. Doi: [10.1186/s12883-020-01793-2](https://doi.org/10.1186/s12883-020-01793-2)

2.	Femi OL, Mansur N. Factors associated with death and predictors of one-month mortality from stroke in Kano, Northwestern Nigeria. Journal of neurosciences in rural practice. 2013;4(Suppl 1):S56. Doi: [10.4103/0976-3147.116460](https://doi.org/10.4103/0976-3147.116460)

Data collection: At what point were patients captured? Were they captured in the emergency department upon admission? were they captured once admitted in the ward? for those in the intervention arm, how soon after being captured did the intensive monitoring start? Was there a difference in care whether a patient who was allocated intensive vs conventional arm eg admission in ICU for any of the groups? is there any justification for excluding other vital paramters from the monitoring eg GCS, NIHSS. Were assessors trained in the assessment of these vital signs?

*Response:* We thank the Reviewer for these excellent questions. Clarifications have been made in the *Randomization and allocation* and *Exposure* sections of the revised manuscript as follows:

- Patients were captured **upon admission to the medical ward after stroke diagnosis was confirmed with imaging**.
- Allocation to either monitoring group was done **immediately after admission**, and monitoring commenced within **30 minutes of randomization**.
- All participants were managed in the **same general medical wards**. None were admitted to ICU, and there was **no difference in level of care between groups**, aside from the frequency of vital signs monitoring.
- **NIHSS scores** were collected at admission and follow-up but were not recorded as part of the *vital signs monitoring schedule*. GCS was not routinely used because NIHSS was preferred for stroke severity classification.
- All data collectors and outcome assessors were **trained medical officers**, certified in the use of the NIHSS and mRS.

Outcome assessment: Ok

*Response*: Thank you.

Statistical analysis: was the chi squared test used in the analysis?

*Response:* Yes. The chi-square test was used to compare categorical variables including demographic, clinical and laboratory characteristics. This has been explicitly mentioned in the *Statistical analysis* section in the revised manuscript.

Results:

Sociodempgraphic and clinical characteristics: Baseline sociodemographic and clinical characteristics were NOT comparable between the two groups for cigarette smoking, DM status. any explanation for this?

*Response:* We agree and have now acknowledged this in the *Results* and *Limitations* sections. These imbalances, despite randomization, could be due to chance in a moderately sized sample. We have noted their potential influence on outcomes and addressed this as a limitation.

It is also interesting to note that ALL patients in this study were CKD patients. This deserves a special mention and explanation

*Response:* We sincerely apologize for the confusion. GFR stages G1 and G2 have now been included in Table 2.

vital signs at 72 hrs: the observable statistical difference in diastolic BP between the 2 groups may reflect a mere numeric relationship. clinically a diastolic blood pressure of 88mmHg and 90mmHg may have not have a significant biological affect on the outcomes. So it may be statistically significant but with no practical clinical implications.

*Response:* We agree with the Reviewer. In the revised *Discussion* section, we have clarified that the importance of this finding lies more in its **association with survival**, rather than in the **numerical BP reduction itself**. (lines 339-347 in the Revised manuscript with track changes)

Stroke severity at 72 hrs; Ok

*Response*: Thank you.

Outcomes at 14 days: ok

*Response*: Thank you.

30 day mortality : ok

*Response*: Thank you.

Discussion: quite brief.

*Response:* The *Discussion* has been expanded to better contextualize the findings, clarify limitations (e.g., imbalanced baseline variables), and discuss implications for stroke care in low-resource settings.

conclusion: ok

*Response*: Thank you.

Recommendations: OK

*Response*: Thank you.

referenecs: Ok

*Response*: Thank you.

the figshare link provided at the end of the manuscript that contains all the raw data is not accessible.

*Response*: Thank you for highlighting this issue. We have removed the inaccessible figshare link. Due to ethical restrictions and conditions of informed consent, the dataset cannot be publicly shared. However, we have updated the Data Availability section to reflect that data will be made available upon reasonable request to the MUHAS IRB via the Corresponding Author. This ensures compliance with institutional policy while supporting transparency.

Generally an important topic but important methodological concerns need to be addressed

*Response:* We appreciate the Reviewer’s recognition of the relevance of our study. We have addressed all methodological concerns raised and revised the manuscript to improve clarity, accuracy, and transparency.

**Reviewer #2**:

This is a very well written and compelling study that supports the establishment of stroke units with advanced monitoring capabilities in resource-limited settings.

We are grateful to Reviewer #2 for the generous and thoughtful feedback. Your kind words and detailed critique have greatly helped us refine and elevate the clarity, accuracy, and presentation of our work. Below, we address each point raised:

**Abstract**:

- In the background, there are a lot of parenthesis which is distracting to the reader. Consider removing the parentheses with outcomes as you can list them below in the methods.

*Response*: We agree. Parentheses have been removed from the Background section of the Abstract, and outcomes are now introduced in a cleaner structure within the Methods section.

- No need to say 306 patients twice, put in results only.

*Response*: This duplication has been removed. The number of participants is now stated only in the Results subsection.

- In the results you mention diastolic blood pressure variability at 72 hours as a main finding but above say you are reporting on outcomes at 30 days. Also, you go beyond simply listing the result and interpret it when you say “underscoring its role in clinical stabilization”; consider removing the interpretation and putting this in the Conclusion instead.

*Response*: Thank you for observing this. The phrase “underscoring its role in clinical stabilization” has been removed from the Results subsection of the Abstract and moved appropriately to the Conclusion.

- First sentence of the Conclusion restates the results almost verbatim.

*Response*: We have revised the first sentence of the Conclusion in the Abstract to avoid repetition and focus on broader interpretation rather than reiterating statistical results.

- Excellent final sentence that makes a compelling argument and summarizes the study findings.

*Response*: Thank you.

**Introduction**:

- This is extremely well written, concise, and beautifully organized. Well done!

*Response*: We are grateful for this encouragement.

Just a few comments to improve it:

- One addition that could be helpful to this section would be listing the vital signs you are referring to. Is it heart rate, blood pressure, oxygen saturation? More?

*Response*: We have added the specific vital signs (blood pressure, heart rate, respiratory rate, oxygen saturation, temperature, and blood glucose) in the Introduction to enhance clarity.

- Lines 66-68 are basically restating what is above and I don’t think they are needed at all. The introduction ended beautifully at line 65 on the previous page. If you really wanted a final sentence remove the repeated info and leave only “…Hospital. The results of this study will contribute evidence-based recommendations for optimizing stroke care in LMICs.”

*Response*: As advised, we have removed the redundant lines and retained only the essential summary sentence, which closes the Introduction more effectively.

**Method**s:

- Was ethical approval obtained from NIMR, the National IRB of Tanzania, in addition to the Research and Publication Committee of Muhimbili? I believe studies in Tanzania need NIMR approval, too.

*Response:* We appreciate the reviewer’s attention to ethical oversight. Ethical clearance for this study was obtained from the Research and Publication Committee of Muhimbili University of Health and Allied Sciences (MUHAS). As the study was not a randomized controlled trial and did not involve external collaborators, additional approval from the National Institute for Medical Research (NIMR) was not mandated under Tanzanian research ethics guidelines. This clarification has been added to the manuscript’s Ethics section.

- Were the abnormal vitals reported to clinicians in real time? (line 77-78) Consider adding this detail.

*Response*: Yes, all abnormal vital signs were promptly communicated to the clinical team. This has been explicitly stated in the Methods section under “Clinical data collection.”

- Lines 84-85. You do not need to re-state that studies indicated improved stroke outcomes as you already built the case well in the Introduction. Please remove.

*Response*: Agreed. This repetition has been removed for conciseness.

- Line 85. Do not say “we wanted to evaluate” but rather say “we evaluated” as it is more direct, crisp writing.

*Response*: Corrected as suggested.

- Line 91-92. Perhaps define stroke mimics and also reliable informant when describing exclusion criteria.

*Response*: Definitions have now been added to the *Study population* subsection of the *Materials and methods* section:

- **Stroke mimics** were defined as clinical syndromes initially suspected to be stroke but later found to be due to other conditions (e.g., brain tumors, metabolic encephalopathies).
- A **reliable informant** referred to a relative or caregiver able to provide accurate clinical and behavioral history for patients unable to communicate due to aphasia or impaired consciousness.

- In Sample Size, why was 35% relative reduction chosen?

*Response*: We thank the reviewer for this important observation. The expected 30-day mortality rates used in our sample size calculation—61% for the 12-hourly group and 37% for the 6-hourly group—were drawn from two prospective cohort studies conducted in similar sub-Saharan African settings (Tanzania and Nigeria, respectively). Comparing these two proportions yields an estimated **39% relative reduction** in mortality.

However, to ensure a conservative and achievable effect size that remained clinically meaningful, we powered the study to detect **at least a 35% relative reduction** in mortality. This approach ensured that our sample size remained robust while grounded in real-world data, and allowed sufficient power to detect a mortality difference likely to influence stroke care strategies in resource-limited settings. We have the revised Sample Size subsection of the manuscript to make this clearer.

- For the randomization section, why was it opened by the participant? In order to take the vital signs, I’m assuming a healthcare provider had to know if they were randomized to one group vs the other so they could take the vital signs on time, so how was it blinded?

*Response*: We thank the reviewer for this important clarification. The randomization envelopes were **not** opened by the participant but by a research assistant in the presence of the participant or their next of kin. This has been corrected in the manuscript.

We initially stated that outcome assessors were blinded to group allocation; however, we now recognize that this was inaccurate. Due to the operational demands of the study and the integration of monitoring frequencies into routine ward care, neither participants, healthcare providers, nor assessors were blinded. We have revised the manuscript accordingly to remove the claim of blinding and clarify the open-label nature of the study. We appreciate the opportunity to correct this and ensure full transparency. A clarifying statement has been added to the *Randomization and allocation* subsection.

- Line 161. How was follow-up performed? By calling participants or home visits or clinic follow-up visits?

*Response*: We thank the reviewer for this important question. All stroke patients remained hospitalized for at least 72 hours post-admission. Follow-up at 14 and 30 days was conducted through scheduled in-person visits at the neurology clinic, in line with clinic protocol. All surviving patients returned for follow-up, ensuring complete outcome data. For participants who did not attend, follow-up was conducted via phone calls to confirm outcomes, including deaths, as reported by relatives or next of kin. This clarification has been added to the *Materials and methods* section.

**Results**:

- Overall excellent job summarizing the pertinent findings from the tables.

*Response*: Thank you.

- Vitals signs at 72 hours by monitoring strategy – Was the intensive monitoring group just sicker, and that is why they had increased mortality? This should be discussed in Limitations or Discussion.

*Response*: We have addressed this point in both the *Discussion* and *Limitations* sections. Although baseline stroke severity was not significantly different, we acknowledge the possibility of unmeasured confounders and the higher prevalence of diabetes and smoking in the intensive group, which may have influenced outcomes.

- There are so many tables and figures. Consider removing Table 5 that talks about outcomes at 14 days, or including it as a supplemental table, since there was nothing significant anyway. The more important results are the 30 day outcomes tables.

*Response*: As advised, Table 5 has been moved to the Supplementary section (S1 Table), with reference adjusted in the text.

- Another way to cut down on the number of tables would be to combine tables 6 and 7 into an outcomes table to include both disability and mortality.

*Response*: Done. We have now merged Tables 6 and 7 into a single comprehensive outcomes table (Table 5) presenting both disability and mortality at 30 days.

**Discussion**:

- I haven’t seen title headings for sections of this Discussion before, and don’t really like them. I’ll leave it up to the editor to decide if they will allow the authors to do this or not, but would advise against it.

*Response*: We have removed all subheadings from the Discussion section to conform to standard style and flow.

- Lines 271-272. I think the interpretation here as the vital signs monitoring reduced diastolic blood pressure is incorrect. The monitoring didn’t reduce the blood pressure, but rather it just was that patients that had more intensive monitoring had lower BP. Please reword this inaccurate sentence. This is also where you can discuss if perhaps the intensive monitoring group were just sicker since they had lower diastolic BP. Also consider adding this to the Limitations.

*Response*: Thank you for pointing this out. The subsection has been rewritten.

- The whole vital signs monitoring paragraph needs to be re-thought as per above.

*Response*: This paragraph has been revised for accuracy, removing speculative causality and focusing instead on observed associations and their implications.

- The discussion of functional disability at 72 hours is good, but it doesn’t seem to be highlighted in the Intro/outcomes as a main outcome. Perhaps add it above. Also, consider discussing functional disability at 30 days.

*Response*: We thank the reviewer for this thoughtful observation. Functional disability was pre-specified as a secondary outcome and is described in the Outcome assessment section of the Materials and methods. In the revised manuscript, we have now expanded the Discussion to include a more comprehensive analysis of functional disability at both 72 hours and 30 days, highlighting the extent of recovery over time and the potential influence of rehabilitation services.

- In the Conclusion paragraph, consider a sentence like that in the abstract that calls for the establishment of stroke units – this was very strong.

*Response:* We thank the reviewer for this valuable suggestion. In response, we have revised the Conclusion paragraph to explicitly include a call for the establishment of dedicated stroke units equipped with standardized monitoring protocols and integrated rehabilitation services. This aligns with the strong statement made in the abstract and reinforces the policy and clinical relevance of our findings. The revised Conclusion now highlights the role of stroke units in optimizing outcomes and reducing preventable deaths in resource-limited settings.

**Reviewer #3**:

We sincerely thank Reviewer #3 for the thoughtful and constructive feedback and insightful recommendations, which have significantly strengthened the clarity and rigor of our work. Below are our point-by-point responses to the issues raised:

This study examines the impact of intensive blood pressure (BP) monitoring in LMICs, particularly in settings with limited stroke care resources and without access to a dedicated stroke unit. There are few clarifications and comments are needed:

1. Please consider performing an adjusted analysis to account for potential confounding factors that could influence the main outcome—30-day mortality. This will help determine whether other variables may have affected the results.

*Response*: Thank you for this valuable suggestion. In response, we performed a multivariable analysis using **modified Poisson regression** to adjust for potential confounders, as 30-day mortality was not a rare event and logistic regression could have overestimated the association. As now presented in the revised Results section and S2 Table, baseline stroke severity (NIHSS ≥16), current alcohol use, heart disease, and age ≥60 years were independently associated with higher 30-day mortality. Importantly, **intensive vital signs monitoring remained independently associated with a 35% reduction in 30-day mortality** (RR: 0.65, 95% CI: 0.53–0.79; P <0.001), even after adjusting for sociodemographic, clinical, and stroke-related variables. We believe this adjustment strengthens the robustness of our findings and interpretation.

These results have been added to the Statistical Analysis and Results sections of the manuscript, and discussed accordingly.

2. It would be helpful to present the trend of BP readings over the 72-hour monitoring period (e.g., every 6 or 12 hours). Reporting this can provide insight into BP variability between groups, which is known to be an important factor in stroke complications.

*Response:* We agree. To address this, we have now included a supplementary figure (S1 Fig) consisting of line graphs showing the **trend of systolic and diastolic BP values** at each time point (6-hourly or 12-hourly) across the 72-hour monitoring window for both groups.

This visualization has been referenced in the *Results* section, and relevant interpretation added to the *Discussion*.

3. If any abnormal BP readings or clinical issues were detected during the monitoring period, please clarify how they were managed. How many patients in each group experienced these issues, and what treatments, if any, were given? what cut-off point value of BP set to start treatment (if needed)

*Response*: We thank the Reviewer for pointing this out. Out-of-range vital sign parameters were promptly communicated to attending clinicians for management, according to the hospital protocol. For patients with hemorrhagic stroke, antihypertensive therapy was initiated if the systolic blood pressure (SBP) exceeded 160 mmHg, with the goal of lowering SBP to <140 mmHg within one hour. In cases where the initial SBP was >210 mmHg, the BP was lowered by approximately 60 mmHg. Blood pressure reduction was achieved primarily through intravenous antihypertensives (labetalol or hydralazine) to ensure rapid and controlled BP lowering and maintained with oral agents. For patients with ischemic stroke, as thrombolysis was unavailable, oral antihypertensive treatment was initiated if SBP exceeded 220 mmHg or diastolic blood pressure exceeded 120 mmHg, aiming to achieve a 15% reduction in BP over the first 24 hours. If BP was below these thresholds, treatment was generally deferred during the acute phase to preserve cerebral perfusion.

A total of 80 participants in the intensive monitoring group triggered BP alerts, compared to 72 participants in the usual monitoring group; this difference was not statistically significant (p = 0.457).

We have added this information to the *Materials and methods* and *Results* sections.

4. Kindly clarify whether BP readings were taken every 6 hours for the full 72 hours or until the patient was discharged, whichever came first.

*Response*: We clarify that **vital signs monitoring (6-hourly or 12-hourly) was conducted for the full 72 hours post-admission since there was no** discharge or mortality within the first 72 hours **post-admission**.

This clarification has been added to the *Exposure* and *Data Collection* sections of the revised manuscript.
